# Time-Dependent Antibacterial Effects of *Citrullus Colocynthis* Seed Extract Compared to Calcium Hydroxide in Teeth Infected with *Enterococcus Faecalis*

**DOI:** 10.30476/dentjods.2023.97670.2026

**Published:** 2024-03-01

**Authors:** Yasamin Ghahramani, Najme Mohammadi, Saman Baghaei, Negar Ghorbani Jahandizi

**Affiliations:** 1 Oral and Dental Disease Research Center, Dept. of Endodontics, School of Dentistry, Shiraz University of Medical Sciences, Shiraz, Iran; 2 Dept. of Pediatric Dentistry, School of Dentistry, Shiraz University of Medical Sciences, Shiraz, Iran; 3 Student Research Committee, School of Dentistry, Shiraz University of Medical Sciences, Shiraz, Iran; 4 Postgraduate Student, Dept. of Endodontics, School of Dentistry, Shiraz University of Medical Sciences, Shiraz, Iran

**Keywords:** Antibacterial, Citrullus colocynthis, Calcium hydroxide, Intracanal medicament

## Abstract

**Statement of the Problem:**

Endodontic efforts are focused on eliminating intracanal pathogens. Applying intracanal medicament for infected teeth is beneficial for achieving better antibacterial effects in endodontic treatments. Different intracanal medicaments should be assessed and compared for this purpose.

**Purpose:**

The aim of this study was to assess the antibacterial efficacy of *Citrullus colocynthis* seed extract comparing to Ca(OH)_2_ on teeth contaminated with *Enterococcus faecalis*.

**Materials and Method:**

In this *in vitro* study, a novel strain of *Enterococcus faecalis* (*Enterococcus spp.* ATCC 19433) yielded from the root canal treated tooth with persistent apical periodontitis. The canals of 78 human single-rooted extracted teeth were contaminated with mentioned strain and treated with *Citrullus colocynthis* essential oil and Ca(OH)_2_ for 1, 7, and 14 days. To determine the chemical composition of the oils, gas chromatography-mass spectrometry (GC–MS) was applied. The percentage reduction from baseline c.f.u./mL values was estimated.

**Results:**

Oleic acid, benzoic acid, and gallic acid were the major contents of *Citrullus colocynthis* essential oil. The c.f.u./mL count decreased considerably as contact duration rose for both medicaments. After 7 days, a statistically significant difference was identified between the medicaments. *Citrullus colocynthis* showed higher antimicrobial efficacy. However, after 14 days, no substantial difference was found.

**Conclusion:**

*Citrullus colocynthis* essential oil, displayed great antimicrobial efficacy against *Enterococcus faecalis* higher than Ca(OH)_2_ over the first week contact period.

## Introduction

Preventing or healing periapical pathos is a goal of root canal treatment. So, most of the efforts are focused on eliminating intracanal pathogens [ 1 ]. However, complete depilation of bacteria is not achievable [ 2 ]. Chemomechanical canal preparation, antimicrobial irrigants, and intracanal medicaments are used to accomplish the mentioned goal. Applying intracanal medicament for infected teeth is beneficial for achieving better antibacterial effects [ 3 ].

*Enterococcus faecalis* (*E. faecalis*) could be considered as one of the major challenges in endodontics due to its high resistance to antimicrobial agents, survival in sophisticated conditions, and potential dentinal tubule invasion. It is particularly found in teeth with persistent apical periodontitis. Consequently, the effective elimination of bacteria demands adequate disinfection [ 4 - 5 ]. 

A popular intracanal medicament is calcium hydroxide [Ca(OH)_2_]. It has excellent antibacterial activity [ 6 - 7 ]. However, when used for an extended period, it negatively affects dentin structure [ 8 ]. Furthermore, it has a limited depth of penetration in dentinal tubules. Additionally, it is ineffective against all species of bacteria and their toxic products. It has been suggested that the rise of pH due to Ca(OH)_2_ intracanal application can play an adverse role by assisting the attachment of bacteria to collagen fibers of dentin, resulting in their survival against disinfection procedures [ 9 - 10 ].

Recently, the use of herbal remedies has gained special attention. Their effective role in treating infectious disease, biocompatibility, and alleviating synthetic antibacterial agents has been researched. Due to all of the mentioned features, the tendency to use them has grown in Endodontics. Consequently, studies on the antibacterial effect of some of these herbs, such as *Syzygium aromaticum* [ 11 ], *Arctium lappa* [ 12 ], *Triphala*, green tea polyphenols [ 13 ], *Morinda citrifolia* [ 14 ], *liquorice* [ 15 ], against *E.faecalis* has been performed. One of these herbs with antimicrobial properties is *Citrullus colocynthis* (*C.colocynthis*). This desert plant has also other common names such as Abu Jahl's melon and bitter apple. It usually grows up in Mediterranean Basin and Asia [ 16 - 17 ]. This fruit has variable components such as saponins, flavonoids, alkaloids, glycosides, and fatty acids [ 18 - 9 ].

The plant's fruit has been used for different purposes, such as jaundice and bacterial infections, due to its anti-inflammatory, antimicrobial, antioxidant, and immunostimulatory features. Other pharmacological effects include anti-oxidant, antidiabetic, analgesic, gastrointestinal, reproductive, and protective features [ 20 - 21 ].

Several studies have confirmed its antibacterial and anti-fungal efficacy on oral microorganisms including *Streptococcus mutans*, *Streptococcus salivarius*, *Lactobacillus acidophilus*, and *Candida. albicans* [ 22 - 23 ].

Another study performed to evaluate antibacterial and antifungal features of the fruit extracts, stem, and leaf of *C.colocynthis* showed that leaf menthol extract was a capable source of antibacterial and antifungal features against Gram-negative and Gram-positive bacteria and fungi [ 24 ].

The presence of bioactive compositions in the leaf, fruits, and seed of *C.colocynthis* makes it an antimicrobial agent against various microorganisms [ 25 - 26 ]. Several studies have been performed approving this favorable potential against *Streptococcus agalactia*, *Streptococcus mutans*, *Escherichia coli*, *Streptococcus agalactia*, *Klebsiella pneumonia*, *Streptococcus pneumonia*, *Proteus mirabilis*, and *Staphylococcus aureus* [ 27 - 28 ]. Another study reported a significant antimicrobial potential against sixteen bacteria [ 26 ].

In this study, the antibacterial efficacy of *C.colocynthis* seed extract was assessed and compared to calcium hydroxide in teeth contaminated with *E. faecalis*. The null hypothesis of this study was that the anti-bacterial efficacy of *C.colocynthis* seed extract was comparable to Ca(OH)_2_.

## Materials and Method

### Sample preparation

The Ethics Committee of Shiraz University of Medical Sciences (IR.SUMS.DENTAL.REC.1401.103) approved this research. Seventy-eight closed apex single-root teeth were selected. There were no sign of root fractures or caries in the roots. High-speed handpiece and fissure diamond burs (DiaDent, Maribor, Slovenia) were employed to decoronate the samples below the cemento-enamel junction. To standardize the samples, they were cut to 13 mm. To measure the working length (WL), a #15 K-file (Mani, Tochigi, Japan) was inserted into the canal, so that the file’s tip was apparent at the apical foramen. The WL was 1 mm shorter than what was determined using a #15 K file. The canals were shaped utilizing ProTaper rotary devices (Dentsply Maillefer, Tulsa, Ok, United States) SX, S1, S2, F1, F2, and F3. The irrigation of canals was performed using 2 mL of 2.5% sodium hypochlorite (NaOCl) (NaOCl, Yekta, PakNam Co., Tehran, Iran). Then specimens were irrigated with 17% ethylene diamine tetra acetic acid (EDTA) (EDTA, Ariadent, Asia Chemi Teb Co., Tehran, Iran) for 5 min and ultrasonic activation, for removal of smear layer, followed by 2 mL NaOCl irrigation [ 29 ] Sterile saline was used as final irrigation. To prevent the bacterial leakage, sealing process of each root’s apex was performed by applying composite resin, while the exterior surface, excluding the access cavity, was coated with epoxy adhesive. 

The study groups (n= 10 in each group) were allocated randomly to the six 24-well cell culture microplates (Corning, Corning, NY, USA) and the six control groups (n= 3).The samples were developed following the research of Abbaszadegan *et al*. [ 29 ].

### Preparation of the selected microorganism

The studied microorganism represented a novel strain of *E. faecalis*, derived from a patient's single rooted tooth that needed retreatment due to previous root canal treatment failure and had persistent apical periodontitis. After isolating the root with a rubber dam, the surface of the tooth was disinfected with 30% H_2_O_2_ and 2.5% NaOCl. After obtaining the sample from the root canal, 5% sodium thiosulphate was used to counteract the disinfectants. Preparation of access cavity and removal of obturation material were performed mechanically using Hedström files without applying chloroform. Saline irrigation of the root canal was performed following WL determination. Then, sterile paper points 1 mm shorter than the canal's apex were inserted to absorb the canal's contents. After transferring the paper points to a tube of sterile brain heart infusion (BHI) medium, they were dissolved in a vortex for one minute. The serial dilution technique with a 10-fold dilution was used to determine the number of EF colonies. A 100 µL aliquot of the suspension was embedded on BHI agar plates (enhanced with 5% defibrinated sheep blood) followed by incubation (37°C; 24 hours). The Gram stain, colony morphology, and catalase reaction were applied to identify individual colonies and carbohydrate fermentation patterns [ 30 ].

Molecular markers were used to establish the species and acquire the sequence of an rRNA subunit. The Ghasemi *et al*. [ 31 ] recommended this protocol to extract the DNA from several bacterial strains by PCR using 16S rDNA as a molecular marker. The forward primer and the reverse primer were used respectively. Using polymerase chain reaction (PCR) and universal primers against the 16S rRNA genes, DNA fragments of roughly 800 base pairs (bp) were obtained from the bacterial strains’ genomic DNA. These fragments were then collected using the Abbaszadegan *et al*. research technique [ 29 ].

### Exposure of the samples to *E. faecalis*

After 48 hours of incubation, the isolated *E. faecalis* colonies were suspended in 5 mL BHI broth. The spectrophotometric turbidity of the cell suspension was adjusted to 6×10^8^ c.f.u./mL (two McFarland standards). Sterile BHI broth exchange in the root canals with bacterial inoculums was performed utilizing sterile pipettes under laminar flow; the negative control groups were excluded. At 37°C and 95% RH, the samples were incubated for 21 days. Every two days, fresh BHI was added to each canal to replace half of the contaminated media in order to sustain bacterial feeding. On the last day, tests utilizing the catalase reaction and gram staining were performed to ascertain the purity of the bacterial culture.

### The preparation process of the medicament

A strict aseptic condition was used for all preparations. To prepare a paste-like consistency, Ca(OH)_2_ powder (Golchai Co., Tehran, Iran) was combined with sterile saline (Darupakhsh, Tehran, Iran).

Due to pilot study performed by the authors on *C.colocynthis* before this research, the results showed that the essential oil at first, and alcoholic and aqueous extracts at the next place had the most anti-fungal and anti-bacterial effects.

*C.colocynthis* extract was gained by using the grinding method. Plants were gathered from Sistan and Baluchistan plateaus in Iran. To produce the excellent form of the *C. colocynthis*, the plant samples were dried by maintaining them at room temperature for three weeks. Then, the powder of the dried plant was prepared. The ground samples (seeds of *C. colocynthis*) were hydrodistillied for three hours to obtain the pure essential oil using the Clevenger-type hydrodistillation apparatus. Anhydrous sodium sulfate was used to eliminate water from the essential oil specimens.

Hydroxyethyl cellulose, which is non-ionic and water soluble, was utilized as a thickening agent in many investigations for gel formation owing to its very static nature [ 32 - 33 ]. The stock solution was stored at 4° in the refrigerator [ 34 ].

### GC–MS of the essential oils

The *C.colocynthis* seed extract was GC-MS analyzed on GC-MS equipment, and Agilent Technologies GC systems - GC-7890A/MS-5975C model (Agilent Technologies, Santa Clara, CA, USA) was used to analyze the bioactive GC-MS by applying the technique proposed by Enemor *et al*. [ 35 ]. The chemical composition of *C.colocynthis* seed extract was identified utilizing GC retention time and matching the spectra with computer software standards data. 

### First microbial sampling from the specimens

According to Abbaszadegan *et al*.'s research [ 29 ], the baseline microbiological examination was performed after 21 days by inserting three sterile paper points (Gapadent Co., Ltd., Tianjin, China) for 1 minute each. *E. faecalis* c.f.u./mL counts and Gram stain and colony morphology were then used to evaluate bacterial growth. (Enterococcus spp. ATCC 19433) 

### Re-preparation of the samples

The canals were irrigated for three minutes with 5mL of sterile saline, followed by 17% EDTA. For the final irrigation, a sterile saline solution was employed. Subsequently, after drying the root canals with sterile paper points, intracanal medicament was applied to the canals [ 29 ].

### Study groups

Depending on the kind of medications and how long the samples were kept in the canals, the microplates containing the samples were separated into four groups at random: (a) groups 1–3 (each group consisted of 10 samples): Ca(OH)_2_ for 24h, 1 week, and 2 weeks; (b) groups 4–6 (each group consisted of 10 samples): *C.colocynthis* essential oil for 24h, 1 week, and 2 weeks; (c) groups 7-9 as positive controls (each group consisted of 3 samples): sterile saline for 24h, 1 week and 2 weeks; (d) groups 10-12 as negative controls (each group consisted of 3 samples): no bacterial contamination for 24h, 1 week and 2 weeks.

Application of Ca(OH)_2_ was performed utilizing a # 30 lentulo spiral into the canals (DiaDent, Almere, the Netherlands). The canals were filled with *C.colocynthis* essential oil using sterile endodontic pressure syringes. Insertion of both medicaments into the root canals to the extrusion point of the medicaments from the access cavity was continued. After eliminating the surplus medicament and inserting sterile cotton pellets into the access cavities, incubation of the samples was carried out at microaerophilic environment (37°C) for the contact duration established for all groups.

### Collection of microbial samples from the root canal system at the determined interval

At predetermined intervals, medicaments were extracted from the canal using #35 K-files and irrigated with 5mL sterile saline. Groups 1–3 and 4-6 were irrigated with 1mL of 0.5% citric acid (Merck, Darmstadt, Germany), followed by 2 mL of sterile saline [ 29 ]. Following the recommended period of incubation with the medicaments, the microbial assessment was conducted by inserting three sterile paper points. *E. faecalis* counts in terms of c.f.u./mL were used to assess bacterial growth.

### Statistical analysis

Initially, the data were logarithmically converted and reported as log_10_(x+10). Afterwards, the percentage of decrease from the baseline c.f.u./mL count in each determined interval (c.f.u.i) was assessed as x= log_10_^c.f.u.t^-log_10_^c.f.u.i/log10 c.f.u.i^ × 100. Then, the percentage decrease comparison was conducted. Non-parametric utilizing the Kruskal–Wallis and Mann–Whitney tests determined the data's normality. Software SPSS version 22 was used for data collection and analysis (IBM Co., Chicago, IL, USA).

## Results

### Targeted microorganism isolation

**Figure 1:**
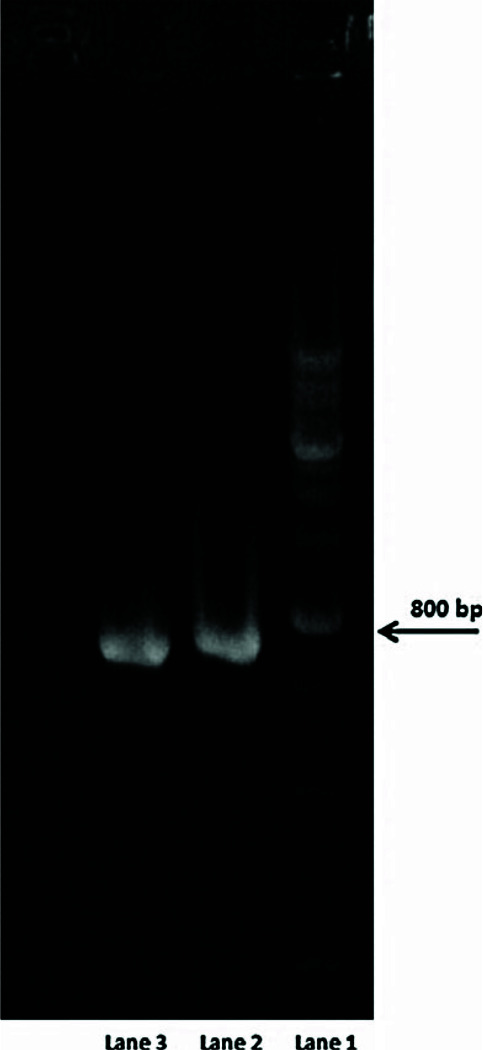
Analysis of 16S rRNA fragments of Enterococcus spp. obtained after polymerase chain reaction amplification

The isolated microorganism was confirmed as b-hemolytic, gram-positive, catalase-negative, and facultative anaerobic cocci. Under UV light, gel electrophoresis results (Figure 1) revealed a band at nearly 800 bp. According to the assessments, there was 98% similarity between the isolate and *E. faecalis*. The strain is currently approved as Enterococcus spp. ATCC 19433.

### GC–MS of the *C.colocynthis* essential oil

The oil isolated by hydrodistillation of *C.colocynthis* resulted in light-colored (light yellow) and rich in essential fatty acids. The detailed mineral and chemical contents of the *C.colocynthis* gaseous oils are discussed in Tables 1 and 2, correspondingly. The main mineral con-16srRNA gene sequence showing the length of the gene at and gel electrophoresis. Lane 1, gene ladder; lanes 2 and 3, approximately 800 base pairstents of *C.colocynthis* seed were Ca, Fe, and Mn. The seed fat of *C.colocynthis* consisted of a high proportion of fatty acids, mainly oleic acid, benzoic acid, and gallic acid.

**Table 1:** Mineral composition of seed extract

Peak no.	Compounds	Area (%)	Kov_ats retention indices
1	Ca	640±126	
2	Cu	7.2±2.8	
3	Fe	13.4±3.3	
4	Mn	311±27	
5	P	37±3.2	
6	K	14±2.4	
7	Zn	1.8±0.3	

**Table 2:** Chemical composition of ethyl acetate extract

Peak no.	Compounds	Area(%)	Kov_ats retention indices
1	Monoacetate	3.5	16.33
2	Carboxylic acid	0.74	18.55
3	Silicic acid	0.63	26.81
4	Ribitol	0.45	27.22
5	Palmitic acid	8.27	27.48
6	Gallic acid	9.83	27.60
7	Benzoic acid	12.32	27.80
8	Ferulic acid	1.47	27.93
9	Oleic acid	40.28	28.00
10	Catechin	3.45	29.38
11	Querectin	3.03	31.58
12	Kaempferol	6.77	32.81
13	Tocopherol	5.98	32.25
14	Tetrasiloxane	1.98	32.25
15	Myricetin	1.27	34.76

### Collection of microbial samples from the root canal system at the determined interval

**Figure 2:**
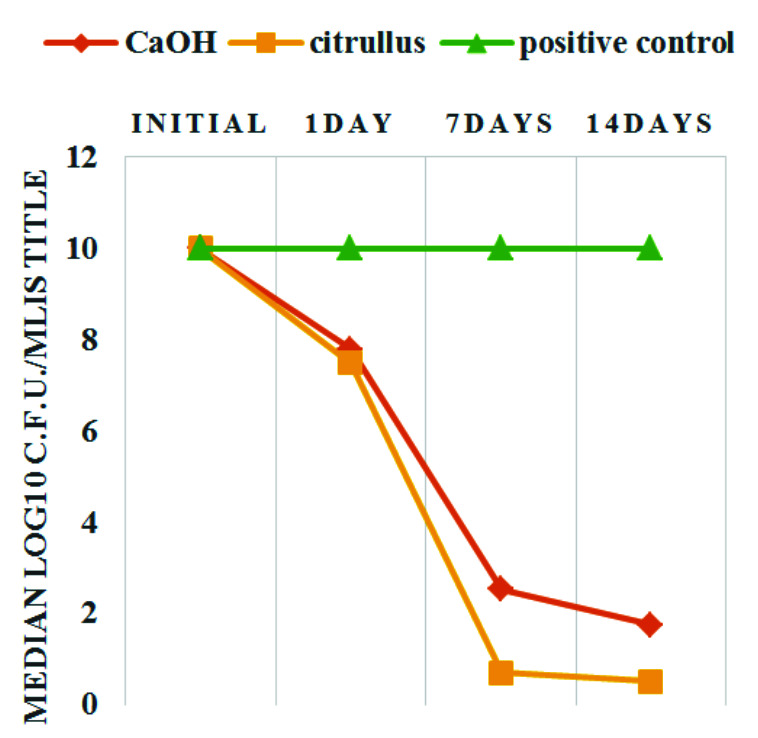
Change in *Enterococcus faecalis* counts (log c.f.u./ mL) by extending the contact time. Bacterial counts in negative controls were zero in all sampling stage

The first sample phase revealed no statistically significant difference in the c.f.u./mL values of the two research groups, as determined by a one-way ANOVA test (*p*= 0.67). The c.f.u./mL values in positive control groups (groups 7-9) exhibited no statistically significant differences during all three periods (*p*= 0.45). In all intervals, the negative control groups (groups 10-12) displayed no microbial growth. The findings verified that both medicaments' c.f.u./mL values declined from baseline levels (Figure 2). By increasing the contact time, the drop percentage of log_10_ c.f.u./mL values were considerably raised in both groups (*p*= 0.00). After one day of incubation, the log_10_ c.f.u./mL value decline was higher in the *C.colocynthis* group than in the Ca(OH)_2_ group, however it was not statistically significant.

Moreover, neither the Ca(OH)_2_ group nor *C.colocynthis* exhibited any significant decrease in bacterial load within 14 days of incubation. A statistically significant difference was noticed between the medicaments after 7 days of incubation not within 14 days. Table 3 presents a summary of the outcomes.

**Table 3:** Median (Mean±Standard deviation) percentage reduction of the log_10_ c.f.u./mL

	1 day	7 days	14 days
Calcium hydroxide	22%(22±2)^a,A^	75%(74±7)^b,A^	85%(85±5)^b,A^
Citrullus colocynthis	25%(25±3)^a,A^	93%(93±4)^b,B^	95%(95±3)^b,A^
Positive Control	1%(1±2)^a,B^	1%(1±2)^a,C^	1%(0±1)^a,C^
Negative Control	0	0	0

† Because the specimens in the negative control groups were not contaminated with bacteria, they were not included in the statistical analyses.

Read vertically, uppercase letters denote comparisons between groups at each defined contact time (adjusted a = 0.013). Read horizontally, lowercase letters denote comparisons between the defined contact times in each group (adjusted a = 0.18). Equal or common letters denote a lack of statistically significant difference (*p*> a).

## Discussion

In the current research, to reconstruct the clinical circumstances of infected root canal systems, samples were contaminated with *E.faecalis*. This species was studied due to its role in the failure of root canal treatment and high resistance capability against Ca(OH)_2_ [ 4 , 9 , 36 ]. The selected bacterium was a novel strain of *E. faecalis* extracted from a tooth exhibiting persistent apical periodontitis that had been endodontically treated earlier. Molecular and culture assessments were confirmed to determine this specimen, which is now known as ATCC 19433.

The effectiveness of Ca(OH)_2_ against *E. faecalis* was assessed, and its antibacterial activity overtime was investigated and compared to *C.colocynthis*. The assessment was made considering that; the amount of medicament contact time in the canal may affect its effectiveness. Nevertheless, the efficacy of Ca(OH)_2_ against *E. faecalis* remains debatable [ 9 ].

No exact time has been determined for the amount needed to achieve the maximum bactericidal effect by medicaments. Behnen *et al*. [ 37 ] declared that the eradication of *E. faecalis* from contaminated canals occurred a day after exposure to Ca(OH)_2_. However, Sjögren *et al*. [ 38 ] declared that remaining Ca(OH)_2_for at least seven days is necessary to show its efficacy. According to several research findings, the Ca(OH)_2_ contact period should exceed 14 days [ 36 , 39 - 40 ]. 

The results of this study demonstrated that even after 14 days, the capability of Ca(OH)_2_ for the complete eradication of *E. faecalis* was deficient. The Ca(OH)_2_ decreased the number of bacterial colonies from 1 to 7 days; however considerable drop in bacterial population did not occur by raising the days to 14 days. Also, studies revealed that even prolonged exposure to Ca(OH)_2_ could not result in a negative culture, which is confirmed with the current data [ 36 , 39 - 40 ].

On the other hand, some of the endodontic literature has shown discouraging results about the antibacterial efficacy of Ca(OH)_2_ against *E. faecalis* due to the buffering action of dentin [ 41 ]. In the study of Vasudeva *et al*. [ 42 ], the dentinal tubule disinfection with Ca(OH)_2_ was less than herbal intracanal medicaments. In this study, *C.colocynthis* extract showed high bactericidal capacity against *E. faecalis*, which was comparable to Ca(OH)_2_ after 1 day. Although the outcomes depended on time that they remained in the root canal, the efficiency of *C.colocynthis* essential oil was comparable to that of Ca(OH)_2_ after one day of incubation. With an increase in contact time, the outcomes for both medicaments were comparable within 14 days.

Up to now, *C.colocynthis* has not been studied as an intracanal treatment against *E. faecalis*. In a study by Seifi Kafshgari *et al*. [ 43 ], the antimicrobial activity of the *C.colocynthis* was evaluated against *Streptococcus mutans* and *Candida albicans*, and showed that *C.colocynthis* could inhibit their growth. Contrary to their study, an *in vitro* study by Gholi *et al*. [ 44 ] showed that the ethanolic and aqueous extracts of *C.colocynthis* showed similar inhibitory roles against *Streptococcus mutans*. Furthermore, none of the extracts demonstrated antibacterial effects on *Lactobacillus acidophilus*. 

Seifi Kafshgari *et al*. [ 43 ] showed that alcoholic and aqueous extracts of *C. colocynthis* fruit pulp had desirable effects on *Streptococcus mutans* and *Candida albicans*. Furthermore, the alcoholic extract suppressed bacterial growth. According to Marzouk *et al*. [ 25 ], the minimal bactericidal concentration (MBC) of *Streptococcus mutans* was inspected in the same concentrations of 1.5mg/mL for both aqueous and alcoholic extracts. This means that the effective concentration of these extracts was proximal.

Marzouk *et al*. [ 25 ] assessed antimicrobial and antifungal features of acetone and aqueous extracts from various parts of *C. colocynthis*, including roots, stems, leaves, and maturation stages of seeds and fruits. The aqueous extracts from the plant were the most effective on *Candida albicans* and *Escherichia coli*. Based on their study, the premature fruit of *C. colocynthis* demonstrated the lowest amount of minimal inhibitory concentration (MIC) against various species of bacteria and fungi.

Bnyan *et al*. [ 45 ] studied the antibacterial efficacy of *C. colocynthis* against several kinds of bacteria. The findings demonstrated that the ethanolic extract inhibited the growth of *Staphylococcus aureus*, *Proteus mirabilis*, and *Escherichia coli*, as well as *Streptococcus agalactia*. In contrast, less or no antibacterial action was exhibited by water extract against all kinds of bacteria. 

The existence of alkaloids, tannins, saponins, steroids, and cardiac glycosides, along with the preliminary phytochemicals, were responsible for the antimicrobial function of the extract of *C. colocynthis* [ 46 ].

*C. colocynthis* is a beneficial plant consisting of medicinally effective components. Different plant parts especially seed extract was an efficient source of the bioactive components with acceptable antimicrobial features. However, more studies should be performed to ascertain the distinct bioactive compounds resulting in antimicrobial features via more advanced techniques [ 47 ]. 

Considering *in vitro* design of this study, further clinical studies should be confirmed to evaluate the clinical antibacterial efficacy of the *C. colocynthis* seed extract as an intracanal medicament.

## Conclusion

Considering the *in vitro* design of the current research, the antimicrobial efficacy of *C. colocynthis* seed essential oil is comparable to Ca(OH)_2_ in infected root canal systems within 14 days. Furthermore, its superior antibacterial efficacy in the first week is an encouraging feature to be considered in clinical conditions.

### Data Availability

The data of this research used to conclude the findings of this study are available from the corresponding author upon request after publication 6–12 months.
